# Comparisons of kinanthropometric variables and physical performance in adolescent soccer players of different biological maturation

**DOI:** 10.7717/peerj.21203

**Published:** 2026-05-13

**Authors:** Rou Wang, Meng Liu, Frank Nugent, Philip Kearney, Indy Man Kit Ho, Cong Li, Hai Pang, Weiming Li, Yaohui Xu, Haochong Liu

**Affiliations:** 1 Sports Coaching College, Beijing Sport University, Beijing, China; 2 Department of Physical Education, University of Jinan, Jinan, China; 3 Physical Education & Sport Sciences, University of Limerick, Limerick, Ireland; 4 Faculty of Management and Hospitality, Technological and Higher Education Institute of Hong Kong, Hong Kong, China; 5 Faculty of Kinesiology, University of Zagreb, Zagreb, Croatia; 6 Facilitate Healthy Developments for Children (Hebei) Technology Co., Ltd., Shijiazhuang, Hebei, China

**Keywords:** Growth, Skeletal maturation, Development, Puberty, Kinanthropometry, Performance

## Abstract

Biological maturation is a crucial factor in the physical development of adolescent soccer players, affecting key performance-related variables essential for talent development. This study investigated differences across maturation stages in kinanthropometric and physical performance parameters in young male soccer players. Forty-three players (13.26 ± 1.03 years) were classified into pre-, circa-, and post-peak height velocity (PHV) groups, with maturation status assessed using a validated AI-based method (China-05). Participants then completed a series of kinanthropometric assessments, including height, body mass, and body composition measured via dual-energy X-ray absorptiometry (DEXA). Additionally, they performed physical performance tests, which consisted of the countermovement jump (CMJ), isometric mid-thigh pull (IMTP), maximal oxygen uptake (VO_2max_) test, Wingate anaerobic test (WAT), 10-meter and 30-meter sprint times, and the Yo-Yo intermittent recovery test (Yo-Yo IR1). Results showed significant differences in fat mass and skeletal muscle mass, particularly between post- and pre-PHV players (*p* < 0.001), with post-PHV players exhibiting higher skeletal muscle mass. In terms of performance, the post-PHV group recorded significantly lower VO_2max_ (*p* < 0.05) but higher CMJ heights (*p* < 0.001), while sprint times showed no significant variation across maturity stages (*p* > 0.05). Fatigue index and peak force also differed notably, with post-PHV players displaying higher fatigue levels and peak force (*p* < 0.001). Significant differences in IMTP impulse at 100 ms and 300 ms (*p* < 0.001) indicated greater strength and power with advanced maturation. These findings underscore the influence of biological maturation on kinanthropometric and physical performance metrics, linking advanced maturation with increased strength and power but reduced aerobic capacity in post-PHV players, likely due to greater body mass. The results highlight the need for tailored training programs that consider both chronological and biological age to optimize player development, supporting more effective talent identification and training strategies.

## Introduction

Soccer is a physically demanding sport that requires a range of technical skills and physical fitness qualities, including muscular strength, speed, aerobic fitness, and anaerobic capacity (Modric, Versic & Sekulic, 2020). In youth soccer teams, these physical attributes are highly valued and players are often selected when they demonstrate superiority over their peers in these parameters (Williams, Ford & Drust, 2020; Xu et al., 2025). The Matthew effect suggests that early-born players who benefit from physical maturity may receive more opportunities, while the Pygmalion effect highlights how coaches’ high expectations for these athletes can enhance their performance (Hancock, Adler & Côté, 2013). Additionally, the Galatea effect demonstrates that athletes’ self-perceptions of competence further drive their success, reinforcing the advantage of early maturation (Hancock, Adler & Côté, 2013). These variations are vastly attributed to the discrepancies in biological maturity status (Söğüt et al., 2019; Massa et al., 2022).

Biological maturation, defined by the rate of progress toward adulthood, involves ‘status’, ‘timing’, and ‘tempo’ (Malina et al., 2015). Maturation varies considerably among young athletes, particularly in boys aged 11–16 and girls aged 10–15, resulting in notable differences in maturity status within the same age group (Rubia et al., 2023). The maturation status of an adolescent athlete refers to the state of physical development at the time of observation, and studies often group youth athletes based on their peak height velocity (PHV) (Malina, Bouchard & Bar-Or, 2004; Albaladejo-Saura et al., 2021; Koziełet al., 2024). These groups are typically categorized into pre-PHV, circa-PHV, and post-PHV, which correspond to the periods before, around and after PHV, respectively. Skeletal age (SA) is regarded as the gold standard for assessing biological maturity, and commonly used methods include Greulich-Pyle (GP), Tanner-Whitehouse (TW) (Tanner, 1962), and Fels (Chumela, Roche & Thissen, 1989), which all share similarities in principle but differ in criteria, procedures, and reference populations. For Chinese adolescents, ethnicity-based differences in growth and development have been identified (Roche, Roberts & Hamill, 1974; Zhang et al., 2008), highlighting the need for population-specific standards. The China-05 bone age standard, based on samples from Chinese pediatric populations and utilizing the TW3 scoring method, has been validated as a reliable tool for this demographic (Pei et al., 2025). In recent years, advancements in deep learning and convolutional neural networks have facilitated the development of artificial intelligence (AI) systems to improve the accuracy of bone age assessments (Prokop-Piotrkowska et al., 2021). AI-driven approaches represent significant advancements in improving the accuracy and reliability of bone age evaluation (Nadeem et al., 2020; Han & Wang, 2020; Liu et al., 2024).

A recent systematic review and meta-analysis of 13 studies highlighted that early maturation in young male athletes showed significantly higher values in kinanthropometric variables (*i.e.,* height, body mass, body mass index (BMI), and body fat percentage) and physical fitness performance (*i.e.,* handgrip strength, countermovement jump (CMJ), squat jump, and medicine ball throw) (Albaladejo-Saura et al., 2021). Despite the evidences on how biological maturation influences physical abilities and kinanthropometric characteristics, research on SA and its association with strength and motor performance in youth athletes remains sparse. Variations in study findings may arise due to differences in study populations, sports, and testing protocols (López-Plaza et al., 2017; Söğüt et al., 2019). Many studies utilize field-based assessments rather than more accurate laboratory methods, often using less precise techniques such as skinfold measurements or bioimpedance instead of the gold standard DEXA for body fat assessment (Tornero-Aguilera, Villegas-Mora & Clemente-Suárez, 2022). Furthermore, the lack of ethnicity-specific skeletal standards constrains our understanding of the maturation–performance relationship across populations (Albaladejo-Saura et al., 2021). Consequently, there remains a critical need for laboratory-validated and population-specific studies to clarify how biological maturation interacts with kinanthropometric and physiological performance indicators in elite youth soccer players.

Therefore, the purpose of this study was to employ the AI-China-05 system to assess the biological maturation status of elite youth soccer players. In addition, comprehensive laboratory-based evaluations of kinanthropometric and physiological variables were conducted to establish precise associations between maturation level and performance characteristics. This integrated and population-specific approach aims to enhance the accuracy of maturation-related performance research and to identify how specific maturity stages correspond to elite soccer performance profiles.

## Methods

### Subjects

Forty-three male elite adolescent Chinese soccer players (chronological age: 13.26 ± 1.03 years; height: 166.46 ± 11.34 cm; skeletal age: 13.76 ± 2.22 years; training experience: 5.59 ± 0.79 years) were recruited from two regional Tier 2 teams (Krustrup et al., 2003) (Table 1). All testing procedures were conducted at the Beijing Sport University (Beijing, China).

The inclusion criteria were: (1) current enrollment in a structured competitive soccer program; (2) age between 12 and 15 years; (3) no musculoskeletal injury or illness within the previous six months; and (4) full availability for all testing sessions. Players with chronic injury, incomplete medical clearance, inconsistent training attendance, or goalkeeper status were excluded to avoid positional bias. The final sample comprised all eligible outfield athletes within the club’s talent development program during the study period; although no formal a priori power analysis was conducted, this cohort represents a typical developmental population, and the unbalanced maturity-group distribution reflects natural biological variation. All participants trained 4–6 times per week (≈2 h per session), focusing on technical–tactical drills, small-sided games, and structured strength and conditioning work. Their weekly schedule also included planned strength and endurance sessions embedded within the periodised training programme. Training content and overall load were consistent across both teams, as verified by their coaches and the regional training-programme curriculum.

**Table 1:** Correspondence between wrist skeletal maturity indices and pubertal growth stages in Chinese adolescent (Zhang et al., 2008).

PHV stage	Puberty growth stage	Indication of wrist skeletal maturity
Pre-PHV	Beginning of growth spurt	Middle phalanx III epiphysis and diaphysis equal in width
	Growth spurt	Middle phalanx III epiphysis squared in shape; sesamoid present
Circa-PHV	Peak height velocity	Middle phalanx III epiphysis covering the diaphysis; sesamoid showing bony knots
	Deceleration of growth	Beginning of fusion of the radial epiphyses; sesamoid matures
Post-PHV	End of growth spurt	Fusion of the epiphysis and diaphysis of middle phalanx III; fusion of one-half of epiphysis and diaphysis of the radius
	Terminal growth	Complete fusion of the radial epiphysis and diaphysis

Written informed consent was obtained from both participants and their parents prior to data collection. Testing was scheduled at least 48 h after competitive matches or high-volume training sessions, and all players completed the full testing protocol. The study was approved by the Research Ethics Committee of Beijing Sport University (Approval number: 2024111H) and conducted in accordance with the Declaration of Helsinki.

### Study design

**Figure 1:**
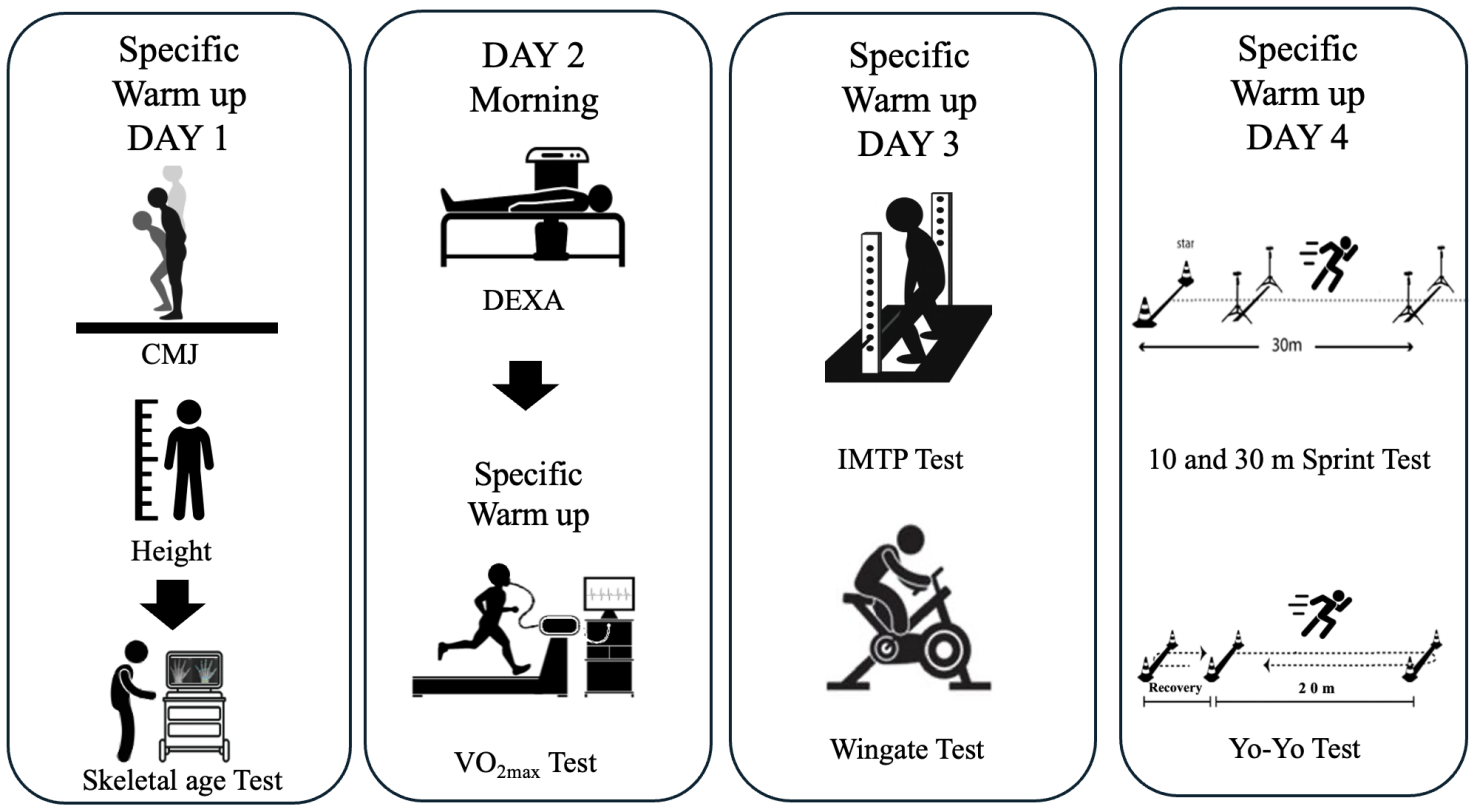
Biological maturation, kinanthropometric, and physical performance parameter test timeline (Albaladejo-Saura et al., 2021). VO_2max_, maximal oxygen uptake; Yo-Yo IR1, Yo-Yo intermittent recovery test level 1; IMTP, isometric mid-thigh pull; Wingate test, Wingate anaerobic test; DEXA, dual-energy X-ray absorptiometry; CMJ, countermovement jump.

The study followed a cross-sectional design with laboratory tests conducted under controlled conditions (22 °C, 50% humidity) and field tests, including the Yo-Yo intermittent recovery test level 1 (Yo-Yo IR1) and sprints, performed on a soccer field. A 48-hour recovery period was provided between the Yo-Yo IR1 and maximal oxygen uptake (VO_2max_) tests. To minimize variability, participants underwent familiarization sessions, and data collection was completed within one week by the same investigator to ensure consistency. The study flow and testing items are shown in Fig. 1.

#### Anthropometrical data

In clinical research, the DEXA test is currently considered the “gold standard” for body composition assessment in clinical research (Marra et al., 2019). For all the participants, body height (cm) was measured barefoot with the head positioned in the Frankfurt plane, using a stadiometer (Seca 264; Seca GmbH & Co. KG, Hamburg, Germany) with a precision of 0.1 cm whereas body composition was measured using GE Lunar anthropometric and body composition scanners (GE Healthcare, Chicago, IL, USA). The measurement parameters were set as follows: length 196.8 cm, width 65.8 cm, exposure factors voltage 100.0 kV, current-15.6 mA, time 7 min 16 s, dose3.0μGv. The test was conducted with whole-body examination and standard mode, and body composition data reports were generated using enCORE™ software (GE Healthcare). Before the test, the operators calibrated the scanner according to the instrument’s operating requirements (Leahy et al., 2012). Participants were instructed to remove clothing, hats, shoes, and socks, as well as any metal objects on their bodies, and lie flat on the scanner with their hands placed at their sides in a still position until notified by the operators that the scan was complete. The observed parameters included body fat percentage (%), fat mass (kg), and muscle mass (kg) for four parts (upper limbs, tight, trunk, and whole body) (Watts, 2004).

#### Isometric mid-thigh pull test

The IMTP protocol was used to assess peak force (PF) during testing. The force plate (Kistler 9286BA) was placed in a custom mid-thigh rack. The subject stood on the force plate, and the bar was adjusted to the upper-middle thigh of the subject. The hip and knee angles were measured using a goniometer, ensuring the hip angle was between 140° and 150° and the knee angle between 135° and 145° (Dos’ Santos et al., 2018). The subject maintained an upright trunk posture with knees slightly flexed. The shoulder girdle was adducted and locked, with feet positioned directly below the bar at hip-width apart. The knees were placed under and slightly in front of the bar, with the thighs lightly touching the bar. The bar position for each subject was recorded to ensure consistency across tests. Subjects used standardized lifting straps to prevent grip failure during testing. For each test, the experimenter issued the standardized instruction: “Push your feet into the ground as fast and as hard as possible”. The experimenter then gave a countdown: “3, 2, 1, pull!” to better prepare subjects for maximal effort. Upon hearing “pull”, the subject performed an all-out isometric pull for five seconds. The experimenter vocally encouraged the subject with phrases like “push” and “exert force” to maximize effort (Dos’ Santos et al., 2017). Timing was controlled by the experimenter, who signaled the subject to stop. Three trials were conducted, with at least 2 min of rest provided between each trial to ensure adequate recovery and consistent maximal effort. The maximum PF recorded was used for analysis. The force-time data during the IMTP were collected using BIOWARE software at a sampling frequency of 1,000 Hz and were processed and analyzed using Microsoft Excel (Comfort et al., 2019).

#### Counter-movement jump

CMJ trials were measured using a high-definition force platform (Hawkin Dynamics Inc., United States; 1,000 Hz) (Badby et al., 2022). Participants performed three CMJs at maximal effort, with a 30-second rest between trials. The average values for jump height and mean power output across the three trials were used for subsequent analysis (Fu et al., 2023). The test has demonstrated excellent test-retest intraday reliability (ICC = 0.906) (Fu et al., 2023).

#### Sprint test

A 10 m and 30 m sprint test were measured by the Smart Speed timing Gates System (Fusion Sport Inc., Brisbane, Australia) as good test-retest inter-day reliability (ICC = 0.881) was found (Fu et al., 2023). The timing gates were placed at the starting point, 10 m and 30 m. Subjects stood at the starting point in a ready position and sprinted to the finish line at full speed after the “3, 2, 1, go” command was given. Participants were instructed to start from a standing start position, without any runs or movements to accelerate before the start line. After two trials, which were performed before and after a 5-minute resting period, the fastest 30 m sprint times were recorded (Fu et al., 2023).

#### Wingate anaerobic test

Anaerobic capacity was measured using the 30 s Wingate anaerobic test (WAT)(Monark894E; Vansbro, Sweden) and peak power (PP), mean power (MP), fatigue index (FI), and time to peak power (TTP) were recorded as parameters of anaerobic exercise capacity. Participant’s height and weight were measured initially, with the load set at 0.075× body weight (kg). Participants engaged in 5–10 min of a standardized warm-up. During the test, resistance was rapidly increased from zero to the predetermined level, and participants pedaled with maximum effort. The bike’s sensors transmitted revolutions per second to a computer, with test personnel providing continuous encouragement to ensure participants maintained maximal effort for 30 s, while ensuring their hips remained in contact with the bike seat throughout the test (Bar-Or, 1987). The reliability coefficients of the WAT for test–retest comparisons (*p* < 0.05) indicate high reliability for PP (*r* > 0.90) (Driss & Vandewalle, 2013) and MP (*r* = 0.91–0.93) (Castañeda-Babarro, 2021). In comparison, the FI exhibits reliability coefficients ranging from 0.43 to 0.73 (Vandewalle, Pérès & Monod, 1987).

#### YO-YO IR1

The test consists of a series of 20 m shuttle runs at progressively increasing speeds, controlled by an audio beep, with regular short rest periods of 10 s (Krustrup et al., 2003). The test was terminated when a participant failed to reach the finish line in sync with the audio beep on two separate occasions. The final distance covered was recorded as the test result. The YY IR1 has been shown to exhibit high reproducibility in youth soccer players aged 13 to 18 years, with intra-class correlation coefficients (ICC) values ranging from 0.87 to 0.95 and coefficients of variation between 3.0% and 7.5%, demonstrating its reliability as a measure of intermittent endurance capacity (Deprez et al., 2015).

#### VO_2max_ test

An incremental step test was conducted using a H/P Cosmos treadmill (Germany) to analyze gas metabolism. Participants performed warm-up exercises for 5–10 min before each test. The modified protocol for measuring VO_2max_ in adolescents began with a warm-up at nine km/h and a 1% incline for three minutes (Hill-Haas et al., 2009). Following the warm-up, the test commenced with a speed increase of one km/h every minute for the first five minutes, maintaining the incline at 1%. Once the speed reached 13 km/h, the speed was kept constant while the incline was increased by 2% every minute until the participant reached voluntary exhaustion. Immediately upon cessation, the time to exhaustion in seconds and the Rate of Perceived Exertion (RPE) using the Borg 6–20 scale were recorded. A heart rate monitor was used to record the heart rate at 5-second intervals throughout the test and at 5-minute intervals during recovery (Liu et al., 2021).

#### Maturation status

##### Interpretation of bone age and sesamoid stage.

**Figure 2:**
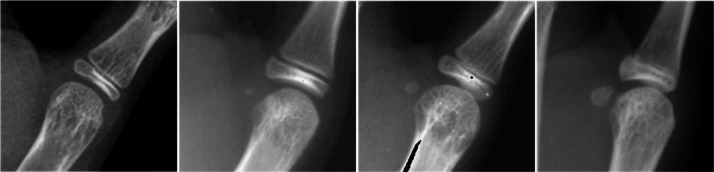
Example stages 0, 1, 2 and 3 of the sesamoid. Stage 1: ossification center present with calcified spots visible and unclear margins; stage 2: osseous node present, well-defined with continuous and smooth margins; stage 3: mature stage characterized by flattening of the surface adjacent to the first metacarpal head and visible reticulated trabecular bone structures (Table 1) (Zhang et al., 2008).

Bone age was interpreted by a clinically proven AI system with 96% accuracy based on the China-05 method (Yin et al., 2022; Zhao et al., 2022), while the development stage of the 13 epiphyses was also output by AI. Each adolescent’s sesamoid development stage was determined by an experienced radiologist, who evaluates more than 10,000 radiographs per year, according to the following criteria. Development of the adductor sesamoid was classified into four stages (0–3) (Fig. 2). Stage 1 is characterized by the appearance of an ossification center, typically observed as calcified spots with indistinct margins. Stage 2 presents as a well-defined osseous node with continuous and smooth margins. Stage 3 represents the mature stage, marked by surface flattening adjacent to the first metacarpal head and the presence of reticulated trabecular bone structures (Zhang et al., 2008).

Considerable individual variation exists in adolescent’s growth, especially in the age of life at which they reach the pubertal growth spurt, and skeletal maturity indices at the wrist are strongly correlated with PHV in adolescent (Grave & Brown, 1976; Hägg & Taranger, 1980), so that the presence of specific maturity indices at the wrist can be used in clinical practice to predict the pubertal growth spurt in children (Zhang et al., 2008) (Table 1).

## Statistical Analyses

Data are presented as mean ± standard deviation. To examine the differences between groups (pre-PHV, circa-PHV, and post-PHV), one-way analyses of variance (ANOVA) were performed followed by Tukey’s *post-hoc* test. Prior to conducting these tests, data were checked for normality using the Shapiro–Wilk test and for homogeneity of variances using Levene’s test. When the assumption of homogeneity was satisfied, standard ANOVA procedures were applied; in cases of unequal group sizes, the Tukey–Kramer adjustment was used for *post-hoc* comparisons. Additionally, effect size (ES) analysis was conducted. Threshold values for Cohen’s d for ES statistics were defined as follows: <0.2 (trivial), ≥0.2–0.6 (small), ≥0.6–1.2 (moderate), ≥1.2–2.0 (large), and ≥2.0 (very large). The level of statistical significance was set at *p* < 0.05 (Bilsborough et al., 2014).

## Results

The study evaluated physical performance metrics across three groups: pre-PHV (*n* = 12), circa-PHV (*n* = 11), and post-PHV (*n* = 20). Body fat percentage showed no significant differences among groups (*p* = 0.27). However, fat mass was significantly higher in post-PHV (9.63 ± 3.22 kg) compared to pre-PHV (6.87 ± 1.02 kg, *p* < 0.001, ES = 1.15, 95% CI [0.19–2.12]). Similarly, skeletal muscle mass was greater in post-PHV (45.67 ± 14.86 kg) than in pre-PHV (29.49 ± 6.57 kg, *p* < 0.001, ES = 1.39, 95% CI [0.4–2.39]), The 10 m sprint times were 2.07 ± 0.10 s for pre-PHV and 1.97 ± 0.15 s for post-PHV (*p* = 0.05), while 30 m sprint times were 4.99 ± 0.25 s and 4.72 ± 0.41 s, respectively (*p* = 0.13). Although a small between-group difference was observed in 10 m sprint performance, this did not reach the predefined significance threshold (*p* < 0.05) and was therefore interpreted as a non-significant trend. VO_2max_ was significantly lower in post-PHV (52.13 ± 6.84 ml min^−1^ kg^−1^) compared to pre-PHV (57.85 ± 5.83 ml min^−1^ kg^−1^, *p* = 0.03), while no significant differences were found between pre- and circa-PHV or between circa- and post-PHV groups (*p* > 0.05).

CMJ height was significantly greater in post-PHV (33.95 ± 1.76 cm) than in both pre-PHV (29.25 ± 4.20 cm, *p* < 0.001, ES = 1.69, 95% CI [0.67–2.72]) and circa-PHV (30.36 ± 2.34 cm, *p* = 0.02, ES = 1.29, 95% CI [0.29–2.30]), whereas no significant difference was observed between pre- and circa-PHV groups (*p* = 0.28). Peak power, mean power, and time to peak power showed no significant differences (*p* = 0.59, *p* = 0.36, *p* = 0.53, respectively).

Fatigue index was significantly higher in post-PHV (71.28 ± 16.92%) than in pre-PHV (56.30 ± 11.87%, *p* < 0.001, ES = 1.12, 95% CI [0.16–2.09]) and circa-PHV (52.64 ± 3.55%, *p* = 0.01, ES = 1.40, 95% CI [0.38–2.41]), while no significant difference was found between pre- and circa-PHV groups (*p* = 0.41). Peak force and relative peak force were significantly greater in post-PHV (peak force: 20.76 ± 3.15 N; relative peak force: 0.31 ± 0.03 N kg^−1^) compared to pre-PHV (peak force: 11.53 ± 1.09 N, *p* < 0.001, ES = 3.16, 95% CI [1.89–4.43]; relative peak force: 0.28 ± 0.01 N kg^−1^, *p* < 0.001, ES = 1.55, 95% CI [0.54–2.56]), but not significantly different from circa-PHV (*p* > 0.05). Impulse at 100 ms and 300 ms (absolute and relative) were significantly greater in post-PHV than in pre-PHV (Impulse 100 ms: 37.37 ± 9.44 N s *vs.* 16.99 ± 2.80 N s, *p* < 0.001, ES = 2.85, 95% CI [1.64–4.06]; Relative Impulse 100 ms: 0.57 ± 0.16 N s kg^−1^ *vs.* 0.41 ± 0.06 N s kg^−1^, *p* < 0.001, ES = 1.31, 95% CI [0.33–2.29]; Impulse 300 ms: 270.52 ± 50.18 N s *vs.* 125.11 ± 12.94 N s, *p* < 0.001, ES = 4.01, 95% CI [2.56, 5.45]; Relative Impulse 300 ms: 4.03 ± 0.47 N s kg^−1^ *vs.* 3.00 ± 0.24 N s kg^−1^, *p* < 0.001, ES = 2.72, 95% CI [1.53–3.9]). No significant differences were found between circa- and post-PHV groups for any impulse measures (*p* > 0.05).

These results highlight significant differences in fat mass, skeletal muscle mass, CMJ height, fatigue index, force production, and impulse generation across maturity groups, with post-PHV individuals demonstrating superior physical attributes in key strength and power measures (Table 2).

**Table 2:** Between group comparisons in the anthropometrical, physiology and physical fitness variables based on the maturity status (mean ± SD).

Variable	Mean ± SD			ANOVA		ES		
	pre-PHV (*n* = 12)	circa-PHV (*n* = 11)	post-PHV (*n* = 20)	*F*	*P*	Pre- Circa	Circa- Post	Pre- Post
Body fat (%)	17.07 ± 2.81	16.11 ± 1.83	15.10 ± 3.75	1.38	0.27	−0.31 (−1.35, 0.74)	−0.32 (−1.27, 0.62)	−0.63 (−1.56, 0.3)
Fat mass (kg)	6.87 ± 1.02	7.90 ± 1.42	9.63 ± 3.22	6.89	<0.001^#^	0.43 (−0.62, 1.48)	0.72 (−0.24, 1.68)	1.15 (0.19, 2.12)
Skeletal muscle mass (kg)	29.49 ± 6.57	37.55 ± 8.52	45.67 ± 14.86	9.58	<0.001^#^	0.69 (−0.37, 1.76)	0.7 (−0.26, 1.66)	1.39 (0.4, 2.39)
10 m sprint (s)	2.07 ± 0.1	2.07 ± 0.11	1.97 ± 0.15	3.23	0.05	0.04 (−1.01, 1.08)	−0.8 (−1.76, 0.17)	−0.76 (−1.7, 0.18)
30 m sprint(s)	4.99 ± 0.25	4.86 ± 0.36	4.72 ± 0.41	2.19	0.13	−0.35 (−1.4,0.7)	−0.4 (−1.35,0.54)	−0.75 (−1.69,0.18)
VO_2max_ (ml min kg)	57.85 ± 5.83	52.83 ± 3.07	52.13 ± 6.84	3.87	0.03^#^	−0.86 (−1.93, 0.21)	−0.12 (−1.06, 0.82)	−0.98 (−1.94, −0.03)
Yo-Yo IR1(m)	2,076.67 ± 419.08	2,098.18 ± 348.42	1,813.00 ± 469.59	2.20	0.12	0.05 (−0.99,1.09)	−0.67 (−1.62, 0.29)	−0.62 (−1.54, 0.31)
CMJ (cm)	29.25 ± 4.2	30.36 ± 2.34	33.95 ± 1.76	14.03	<0.001^†^^#^	0.4 (−0.65,1.45)	1.29 (0.29,2.3)	1.69 (0.67,2.72)
PP (W/kg)	9.56 ± 0.53	9.86 ± 0.75	9.54 ± 1.07	0.53	0.59	0.34(−0.7,1.39)	−0.37(−1.31,0.57)	−0.03(−0.94,0.89)
MP (W/kg)	4.31 ± 1.38	4.69 ± 1.17	4.90 ± 0.91	1.04	0.36	0.34(−0.71,1.39)	0.19(−0.75,1.13)	0.53(−0.4,1.45)
TPP (s)	4.43 ± 2.06	3.86 ± 2.27	3.72 ± 0.66	0.66	0.53	−0.35 (−1.4,0.7)	−0.09 (−1.03,0.85)	−0.44 (−1.36,0.48)
FI	56.30 ± 11.87	52.64 ± 3.55	71.28 ± 16.92	11.10	<0.001^†^^#^	−0.28 (−1.32,0.77)	1.4 (0.38,2.41)	1.12 (0.16,2.09)
Peak force (N)	11.53 ± 1.09	16.52 ± 3.73	20.76 ± 3.15	73.56	<0.001^†^^#^^*^	1.71 (0.56,2.86)	1.45 (0.43,2.47)	3.16 (1.89,4.43)
Relative peak force (N kg^−1^)	0.28 ± 0.01	0.29 ± 0.01	0.31 ± 0.03	9.14	<0.001^#^	0.81 (−0.26,1.88)	0.74 (−0.22,1.7)	1.55 (0.54,2.56)
Impulse 100ms (N.S)	16.99 ± 2.80	24.15 ± 5.11	37.37 ± 9.44	33.01	<0.001^†^^#^	1.00 (−0.08,2.08)	1.85 (0.78,2.92)	2.85 (1.64,4.06)
Relative impulse at 100 ms (N s kg^−1^ )	0.41 ± 0.06	0.48 ± 0.09	0.57 ± 0.16	8.46	<0.001^#^	0.56 (−0.49,1.62)	0.74 (−0.22,1.7)	1.31 (0.33,2.29)
Impulse 300ms (N.S)	125.11 ± 12.94	176.24 ± 17.24	270.52 ± 50.18	90.16	<0.001^†^^#^^*^	1.41 (0.29,2.52)	2.60 (1.41,3.79)	4.01 (2.56,5.45)
Relative impulse 300 ms (N s kg^−1^)	3.00 ± 0.24	3.49 ± 0.31	4.03 ± 0.47	28.30	<0.001^†^^#^^*^	1.30 (0.19,2.4)	1.42 (0.4,2.44)	2.72 (1.53,3.9)

**Notes.**

* Significant difference (*p* < 0.05) between pre-PHV and circa-PHV Group.

# Significant difference (*p* < 0.05) between pre-PHV and post-PHV.

† Significant difference (*p* < 0.05) between circa-PHV and post-PHV Group.

PHV, Peak Height Velocity; pre-PHV, Pre-Peak Height Velocity; circa-PHV, Circa-Peak Height Velocity; post-PHV, Post-Peak Height Velocity; ANOVA, Analysis of Variance; ES, Effect Size; CM, Centimeter; kg, Kilogram; VO_2max_, Maximal Oxygen Uptake (ml min^−^^1^ kg^−^^1^); Yo-Yo IR1, Yo-Yo Intermittent Recovery Test Level 1 (m); CMJ, Countermovement Jump (cm); PP, Peak Power (W kg^−^^1^); MP = Mean Power (W kg^−^^1^); TPP, Time to Peak Power (s); FI, Fatigue Index (%); N, Newton (Force); IMTP, Isometric Mid-Thigh Pull; Relative Peak Force, Relative Peak Force (N kg^−^^1^); Impulse 100 ms, Impulse at 100 Milliseconds (N s); Relative Impulse 100 ms, Relative Impulse at 100 Milliseconds (N s kg^−^^1^); Impulse 300 ms, Impulse at 300 Milliseconds (N s); Relative Impulse 300 ms, Relative Impulse at 300 Milliseconds (N s kg^−^^1^).

## Discussion

The present study examined the impact of biological maturation on the kinanthropometric and physical performance parameters of adolescent Chinese soccer athletes. The findings indicate that there are significant differences across multiple kinanthropometric and physical performance parameters (fat mass, skeletal muscle mass, VO_2max_, CMJ height, fatigue index, IMTP peak force, and impulse at 100 ms and 300 ms), among pre-PHV, circa-PHV, and post-PHV groups. Despite the comparable body fat percentage across different maturation stages, significant differences were observed in fat mass and skeletal muscle mass, with post-PHV individuals showing higher values in both metrics. Similarly, athletes in the post-PHV group were significantly older, taller (height), and heavier (weight). The larger body figure, longer lever arm, higher moment of inertia, and the skeletal muscle size would potentially give additional advantage in strength (force output) and power (explosive strength) development as previous research has established a strong relationship between increased muscle mass and enhanced power output (Moritani & DeVries, 1979; Petré, Wernstål & Mattsson, 2018).

In this regard, testosterone, an indicator of the hypothalamic–pituitary–gonadal axis, plays a crucial role in advancing puberty and significantly increases during adolescence in males, sometimes up to 30 times higher in the post-adolescence stage (young adults) (Handelsman, Hirschberg & Bermon, 2018). Testosterone plays a pivotal role in strength and power development through its anabolic effects on lean muscle mass. Although the role of testosterone in muscle development is well established, previous studies relying on relative percentage measurements may have overlooked significant variations in muscle mass. In this regard, López-Plaza et al. (2017) demonstrated that absolute muscle mass and force output differ significantly across maturation stages, with post-PHV athletes exhibiting greater muscle mass compared to pre-PHV and circum-PHV groups, which may confer a competitive advantage in specific sports or playing positions. Additionally, other body masses, such as fat mass, can increase during growth periods, potentially masking changes in muscle mass when relative mass is used for observation. Valente-dos-Santos et al. (2012) assessed fat-free mass by subtracting fat mass from total body mass. Since fat-free mass includes various tissues such as bone, skin, and visceral mass, this method can underestimate the changes produced in muscles. Our use of DEXA, which provides precise measurements of muscle mass in kilograms, overcomes these potential limitations and offers a more accurate understanding of the relationship between muscle mass and athletic performance (Bilsborough et al., 2014). This approach reveals nuanced insights into how variations in muscle mass contribute to performance disparities observed among different maturation groups.

Our study revealed significantly higher PF and relative PF in the post-PHV group, aligning with previous research that maturation enhances absolute strength capabilities in youth soccer players (Morris et al., 2020). This enhancement is due to physiological growth and muscle development during puberty, driven by increased anabolic hormones like testosterone, which facilitate muscle hypertrophy and neuromuscular efficiency (Fink, Schoenfeld & Nakazato, 2018). Furthermore, we observed significant differences in absolute and relative impulse at 100 ms and 300 ms, supporting findings by Emmonds et al. (2017) that mature athletes exhibit greater impulse generation (Emmonds et al., 2017). This is likely due to improved neuromuscular coordination and muscle–tendon stiffness, which enhance force transmission efficiency and the ability to store and release elastic energy. Although there was no clear advantage in 10- and 30-m sprint performance, the post-PHV group showed potentially faster 10-m sprint speeds. This suggests that factors like longer limbs, providing a mechanical advantage by increasing force application distance, play a significant role. Additionally, the hormonal surge during puberty improves neuromuscular signaling efficiency, facilitating faster and more coordinated muscle contractions (Brownlee et al., 2018). These results reveal the complex interplay of hormonal changes, neuromuscular adaptations, and biomechanical factors in enhancing physical performance during maturation.

In addition to the clear advantages in absolute strength and impulse with maturation, post-PHV also outperformed pre-PHV in several relative strength metrics (*e.g.*, relative PF and impulse). It is believed that apart from the strength development issues, other factors, such as the different training experience and current strength training practices within youth soccer environments, can also contribute to the observable relative strength deficits (Emmonds et al., 2017). Given such relative strength deficits, specific training programs for enhancing relative strength are needed. Exercises targeting rapid force production, such as plyometrics and high-intensity interval training, could be particularly beneficial (Hughes, Ellefsen & Baar, 2018; Comfort et al., 2024). Furthermore, the significant interindividual variability in IMTP characteristics within the same chronological age groups highlights the importance of personalized training programs. This variability can be attributed to differences in maturation, training age, physical abilities, and individual responses to training (Brownlee et al., 2018). Therefore, practitioners should assess the maturity status of individual athletes to design tailored strength and conditioning programs that cater to their specific developmental needs. Overall, the findings underscore the importance of considering both absolute and relative measures of strength and impulse when evaluating the physical capabilities of adolescent youth soccer players. Future research should explore force-time-specific values at different times to provide a more detailed understanding of an athlete’s force-generating capabilities and further refine training practices to enhance both absolute and relative strength measures.

Anaerobic performance assessed by the Wingate test did not show a significant difference among the three groups in our study which is an interesting finding. This outcome provides valuable insights into the development of anaerobic capacity during adolescence. Previous studies have reported that anaerobic performance is influenced by muscle mass, glycolytic enzyme activity, and neuromuscular coordination, all of which can vary with growth and development (Armstrong & Welsman, 2007; Armstrong & Welsman, 2020). However, the recent cross-sectional study by Armstrong & Welsman (2020) only investigated the anaerobic performance distribution across the ages of 11 to 18 without assessing the biological maturation of each participant. Given that muscle mass and neuromuscular efficiency improve with age, one might expect post-pubertal athletes to perform better. Nonetheless, the results of the current study suggest that the anaerobic capacity of younger athletes may be sufficiently developed to match that of their older counterparts, possibly due to early sports specialization and training regimens (Eisenmann, Till & Baker, 2020). The similar performance across maturity stages might also be attributed to the training backgrounds of the participants. Athletes involved in regular high-intensity training from a young age often develop significant anaerobic capacity regardless of their maturity stage as all three groups showed no difference in training experience. Specifically, pre-PHV adolescents may require more sprinting during training and competition to keep up with their post-PHV counterparts, thus better developing their anaerobic capacity (Abbott et al., 2019). Training-induced adaptations, such as increased phosphocreatine stores, enhanced glycolytic enzyme activity, and improved neuromuscular coordination, could contribute to the observed uniformity in Wingate test results (Vera-Ibañez et al., 2017).

In terms of 10 m sprint performance, our current findings indicated a non-significant difference (*p* = 0.05). This marginally non-significant result may be partly attributed to the small sample size, sampling variations, and individual differences. Likewise, Yang & Chen (2022) reported that among 14- and 15-year old soccer players, the early-maturing group did not clearly outperform their late-maturing counterparts. Interestingly, the early-maturing players in their younger group, who were the same age as our athletes (13-year-olds), clearly sprinted faster than the late-maturing players, yielding different results from ours. Our post-PHV group demonstrated distinct advantages in several strength-related metrics including CMJ height, skeletal mass, peak and relative peak force, impulse measures at 100 ms, and 300 ms. However, the muscle mass and strength-related metrics were not clearly assessed in their study. Given that 10 m sprint performance is closely associated with explosive strength, it remains inconclusive whether biological maturation provides a definitive advantage in this aspect of starting acceleration without further comparisons of pre-PHV and post-PHV strength and 10 m sprint performance in larger samples from diverse populations. On the other hand, it is evident that 30 m sprint performance did not differ significantly across maturity groups, aligning with findings for 15-year-old players in the previous study (Yang & Chen, 2022). However, similar to the 10 m sprint results, our study also revealed some discrepancies with their findings, particularly in the 13- and 14-year-old groups. This suggests that short-distance sprint ability is not solely driven by biological maturation but can also be strongly influenced by other factors such as neuromuscular coordination, technique, and acceleration mechanics. Additionally, the participants had comparable training experience and were regularly exposed to high-intensity training from an early age, which may have contributed to similar levels of anaerobic capacity and sprint performance across groups (Eisenmann, Till & Baker, 2020). Therefore, training-related adaptations may have overshadowed potential maturation-related differences in sprint outcomes. Since it was speculated that plyometric training and the combined training methods could have different effects on pre-, mid-, and post-PHV highlighted in the previous study (Rumpf et al., 2012), without fully understanding the training history and profile of participants in our study, it seems that sprint speed, a crucial component of many sports, may be influenced by maturity stages differently. Apart from the strength and power output, sprinting techniques related to their training experience might also substantially contribute to their comparable sprinting performance. Therefore, it is speculated that sports skills training or coordination, such as sprinting techniques, should be an area of focus for pre-PHV athletes to minimize their performance deficits when compared to their older counterparts due to physiological differences.

The results of this study demonstrate that pre-PHV soccer players outperformed their PHV and post-PHV groups in both VO_2max_ and YOYO tests. The superior performance of pre-pubertal athletes in VO_2max_ tests can be attributed to their lower body weight and higher relative aerobic capacity. Despite the increased body weight in post-pubertal athletes, this additional weight likely contributes to a greater mechanical load during physical activity, resulting in decreased relative aerobic capacity. The lighter body weight of pre-pubertal athletes may confer an advantage in energy expenditure and oxygen utilization efficiency, thereby enhancing their performance in VO_2max_ tests (Beunen & Malina, 1988; Bacon et al., 2013). Moreover, better YOYO performance in pre-pubertal athletes might be explained by superior neuromuscular control and higher efficiency in lower limb muscle utilization. Pubertal and post-pubertal athletes may experience decreased synchronization of muscle group actions, such as co-contractions, leading to higher mechanical costs during running and other high-intensity activities (Moran et al., 2018). Furthermore, the higher aerobic fitness observed in pre-pubertal athletes may be linked to their longer training history and greater exposure to intermittent physiological loads during training and competition. On the field, specific positional demands and physical attributes required by soccer players vary with maturity stages. Shorter, pre-pubertal athletes may engage more in full-field drills and frequent sprints, whereas taller, post-pubertal athletes typically participate in contact-based situations near the goal area. This positional specialization might limit the cardiovascular conditioning opportunities for post-pubertal players, contributing to their lower aerobic performance (Sweeney et al., 2023). These results emphasize the need for training programs tailored to the maturity stages of young athletes. Such programs should aim to maximize the advantages of each maturity stage while addressing the specific needs and limitations associated with each phase (Jayanthi et al., 2022).

Collectively, the variance in biological maturity accounts for only a part of the story. Contrary to expectations, post-PHV players do not always significantly outperform their pre-PHV counterparts. Our findings, in conjunction with previous research, indicate that pre-pubertal athletes can excel in aerobic capacity and high-intensity activities that require efficient energy metabolism and muscle utilization. In contrast, post-pubertal athletes generally perform better in activities requiring greater muscle mass and strength (Yang & Chen, 2022; Aouichaoui et al., 2024). Additionally, the prior study has reported mixed outcomes regarding early- and late-maturing players across different age groups (Yang & Chen, 2022). Similarly, a recent study involving English Premier League soccer players aged between 11.3 and 16.2 found only weak (height and weight) to moderate (sprint, change of direction, and CMJ performance) correlation between the maturity levels and selected metrics in their linear regression models (Parr et al., 2020). Notably, the reactive strength index exhibited nearly zero correlation in their findings. Together with the results of the current investigation, it appears that biological maturity has a lesser impact on plyometrics tasks, especially those involving fast stretch-shortening cycle (SSC), such as sprinting, compared to other ballistic tasks that demand greater force output, like the CMJ. Early- or late-maturing athletes can leverage their kinanthropometric and physical characteristics to train and develop strategically.

A novel aspect of this study is the introduction of an AI-based system for assessing maturation status, which has been employed for the first time to compare kinanthropometric and physical performance parameters. This system provides a more precise and automated assessment of maturation stages, offering deeper insights into the biological development of athletes. By applying AI to maturation analysis, the objectivity of the assessment is enhanced, potentially reducing observer-related variability that traditional methods often encounter.

While the study provides preliminary evidence linking biological maturation to physical and kinanthropometric characteristics, the findings should be interpreted cautiously due to the limited and unbalanced sample. In particular, the non-significant results observed for sprint and Wingate performance may be partly attributed to the small sample size—especially the reduced number of players in the circa-PHV group—which likely limited the statistical power to detect meaningful differences. Moreover, as this research employed a cross-sectional design, the observed associations should not be interpreted as causal; rather, they indicate correlational relationships that warrant further longitudinal investigation.

## Conclusions

The study examined the impact of biological maturation on adolescent soccer athletes, revealing significant differences in kinanthropometric and physical performance metrics among pre-PHV, circa-PHV, and post-PHV groups. Key findings indicate that maturation is associated with greater fat mass, skeletal muscle mass, and improved strength and power outputs, while relative strength remains unchanged. These results highlight the importance of incorporating relative strength development into youth training programs. Anaerobic performance and sprint speed did not significantly differ across maturity stages, suggesting that consistent training exposure may mitigate physical maturity differences. Pre-PHV athletes showed higher relative aerobic capacity, likely due to lower body mass and greater movement efficiency.

From a practical standpoint, biological maturation should guide training and evaluation in youth soccer. In terms of talent identification, players of post-PHV possess a distinct advantage in the physical qualities mentioned earlier. Consequently, selection processes that primarily emphasize physical fitness performance are likely to favor post-PHV players while overlooking the potential of the pre- and circa-PHV players. Coaches should take these factors into account and explore other dimensions, such as growing potential and adaptability in technical, tactical, and mental areas, to ensure they do not miss out on late-maturing talent. From a training perspective, post-PHV players demonstrate greater absolute strength, which is beneficial for contact and explosive actions, whereas pre-PHV players exhibit superior relative aerobic capacity. The lack of differences in sprinting and anaerobic performance suggests that structured training may partially mitigate the effects of maturational disparities. Meanwhile, coaches may need to adjust the metabolic training program to counteract the potential decline in aerobic capacity for post-PHV players. Consequently, maturity-sensitive, individualized programs are essential to optimizing long-term athletic development and preventing premature talent deselection. For instance, late-maturing players identified as having kinanthropometric and physical disadvantages may enhance their sprinting and soccer-specific performance by improving movement efficiency, neuromuscular coordination, and technique.

Overall, the findings underscore the practical need for individualized, maturity-sensitive training strategies that align both biological and chronological age to optimize youth soccer performance.

## Supplemental Information

10.7717/peerj.21203/supp-1 Supplemental Information 1 Data Raw measurements of kinanthropometric variables and physical performance tests, including participant ID, maturation status, and all test results used for statistical analysis.
数据（全） = ”Data (All)”

## Additional Information and Declarations

### Data Availability

The following information was supplied regarding data availability: The data is available in the Supplemental File.
